# The association between food insecurity and academic achievement in Canadian school-aged children

**DOI:** 10.1017/S1368980017001562

**Published:** 2017-07-20

**Authors:** Erin L Faught, Patty L Williams, Noreen D Willows, Mark Asbridge, Paul J Veugelers

**Affiliations:** 1 Population Health Intervention Research Unit, School of Public Health, University of Alberta, 3–50 University Terrace, 8303-112 Street, Edmonton, AB, Canada, T6G 2T4; 2 Food Action Research Centre (FoodARC), Department of Applied Human Nutrition, Mount Saint Vincent University, Halifax, NS, Canada; 3 Deparment of Agricultural, Food, & Nutritional Science, University of Alberta, Edmonton, AB, Canada; 4 Department of Community Health and Epidemiology, Department of Emergency Medicine, Dalhousie University, Centre for Clinical Research, Halifax, NS, Canada

**Keywords:** Food insecurity, Socio-economic inequalities, Academic achievement, Children

## Abstract

**Objective:**

Education is a crucial social determinant of health. Food insecurity can be detrimental to children’s academic achievement, potentially perpetuating a cycle of poverty and food insecurity. We aimed to assess the relationship between food insecurity and academic achievement in Canadian school-aged children.

**Design:**

Cross-sectional study of children and parents. Parents completed the short-form Household Food Security Survey Module and questions about income and education level (socio-economic status). Children completed FFQ. Data were prospectively linked to children’s performance on standardized exams written one year later. Mixed-effect logistic regression was employed to assess the relationship between food insecurity and likelihood of meeting academic expectations adjusting for socio-economic status, diet quality and potential confounders.

**Setting:**

Nova Scotia, Canada in 2011–2012.

**Subjects:**

Students (*n* 4105) in grade 5 (10–11 years; 2167 girls) and their parents.

**Results:**

Low food security was reported by 9·8 % of households; very low food security by 7·1 % of households. Students from low-income households and reporting poor diet quality were less likely to do well in school. Children who lived in households reporting very low food security had 0·65 times the odds (OR=0·65; 95 % CI 0·44, 0·96) of meeting expectations for reading and 0·62 times the odds (OR=0·62; 95 % CI 0·45, 0·86) of meeting expectations for mathematics.

**Conclusions:**

Very low household insecurity is associated with poor academic achievement among children in Nova Scotia.

Socio-economic status is the most universal determinant of health status in populations^(^ ^1^ ^)^. As socio-economic status increases, so does the likelihood of better health throughout the lifespan^(^ ^1^ ^)^. One of the mechanisms by which low socio-economic status can be detrimental to health is through the development of household food insecurity^(^ ^2^ ^)^. In developed countries, food insecurity refers to inadequate household access to food due to financial constraints^(^ ^3^ ^)^. In Canada, the overall prevalence of household food insecurity is 12·0 % and no statistically significant decreases have been seen since rigorous measurement of Canadian household food insecurity began in 2005^(^ ^4^ ^)^. Given the established profound negative associations food insecurity has with health and well-being outcomes, including poor diet, overweight and obesity, poor childhood development, poor psychosocial functioning and adverse mental health^(^ ^5^ ^–^ ^8^ ^)^, strategies to address this concern are imperative.

For children, good education is one means to leave poverty and to thrive through the remainder of their lifespan^(^ ^9^ ^,^ ^10^ ^)^. However, studies have revealed that food insecurity in childhood is associated with detrimental impacts on healthy development and thus academic success in childhood may be negatively affected, allowing poverty and consequent food insecurity to persist into subsequent generations^(^ ^9^ ^)^. Many studies have been conducted to investigate the association between food insecurity and academic achievement, and results are largely consistent to suggest a negative association between food insecurity and children’s school performance^(^ ^11^ ^–^ ^15^ ^)^.

Our objective was to provide further support for findings associating food insecurity and poor academic achievement among children. We aimed to address this objective using a large, population-based health survey of grade 5 (10–11-year-old) children in Nova Scotia, Canada, prospectively linked to results from exams written one year later. Studies investigating the association between food insecurity and academic achievement are few in Canada. Nova Scotia has been shown to have one of the highest prevalences of food insecurity of all of the provinces (not including the territories) in Canada at 15·2 %^(^ ^4^ ^)^, 32 % of food bank users in Nova Scotia are children^(^ ^16^ ^)^, and food insecurity among Nova Scotian children has been shown to contribute to poor diet quality and a higher BMI^(^ ^17^ ^)^. Given this, further and local evidence on the potential negative association between food insecurity and children’s outcomes in Nova Scotia is important to motivate interventions to address the high prevalence of food insecurity. In our analysis, we also considered other contributors to academic achievement including diet and other lifestyle behaviours^(^ ^18^ ^)^, parental education^(^ ^19^ ^)^ and household income^(^ ^20^ ^)^. We hypothesized that food insecurity would be negatively associated with children’s academic achievement.

## Methods

We used data from the Children’s Lifestyle and School performance Study (CLASS) II conducted in Nova Scotia, Canada, in the spring of 2011. CLASS was a population-based survey aiming to evaluate the lifestyle behaviours, health and school performance of grade 5 students (10–11-year-old children). All schools with grade 5 classes in Nova Scotia were invited to participate and, as such, all members of the population of interest were included in the sampling frame. Two hundred and sixty-nine (94·1 %) school administrators of 286 eligible schools agreed to their school’s participation. Once a school agreed to participate, parents or guardians of all grade 5 students received a home package for their completion. This package contained a consent form allowing the child’s participation in the study and a home booklet directed at the parent containing questions concerning household food security, socio-economic status, demographic factors and other questions related to the home environment and the child’s behaviours. Parents were also asked for consent to link their child’s survey information to their performance on standardized provincial exams they would write in grade 6, one full year following the measurement of exposures of interest and potential confounders.

Trained research assistants travelled to participating schools and administered surveys to children who had returned a signed consent to participate. Students completed two surveys: (i) a student survey developed by the CLASS research team containing questions about their lifestyle behaviours and self-perceptions; and (ii) the Harvard FFQ for Children and Youth (YAQ) adapted for Canadian use^(21)^. Parental consent to participate in the survey was provided for 5913 students, with an average response rate of 67·7 % per school (ranging from 17 to 100 % response). Participants were further excluded from analysis if consent was not obtained for an academic linkage or if the linkage was not successful, if their energy intake was <2092 kJ/d (<500 kcal/d) or >20 920 kJ/d (>5000 kcal/d)^(^ ^22^ ^)^, or if other relevant data were missing. Following all exclusions, data were used from 4430 participants with Reading results, 4625 participants with Writing results and 4310 participants with Mathematics results, giving an overall completion rate of 74·9, 78·2 and 72·8 %, respectively; 4105 (69·4 %) participants had a successful linkage with all three subject areas.

### Food insecurity

Parents of participating students also completed a survey. The US Department of Agriculture Household Food Security Survey Module (HFSSM) is a validated tool that is widely used to assess the prevalence of food insecurity in populations as well as trends in prevalence of food insecurity over time. To reduce respondent burden, household food security was assessed using the six-item short-form HFSSM^(^ ^23^ ^)^. Although less precise and somewhat less reliable than the eighteen-item measure, it avoids asking questions about children’s food security, which can be sensitive in some survey contexts^(^ ^23^ ^)^. It has been shown to be reliable and valid by correctly identifying, with respect to the longer-form eighteen-item questionnaire, the food insecurity classification of 97·7 % of households in an effectiveness study^(^ ^24^ ^)^. However, it cannot be used to identify the most severe forms of food insecurity where children may be experiencing hunger^(^ ^23^ ^)^. The item was scored as provided in the guide and households were categorized as high or marginal food security (zero or one affirmative response), low food security (two to four affirmative responses) and very low food security (five or six affirmative responses). Unlike the eighteen-item HFSSM, the six-item survey does not measure the most severe range of adult food insecurity, in which children’s food intake is likely to be reduced.

### Socio-economic status

Household income and parental level of education were assessed using two questions on the home survey. Possible responses to ‘What is your current household income from all sources?’ were ‘Less than $20 000’, ‘$20 001 to $40 000’, ‘$40 001 to $60 000’, ‘$60 001 to $80 000’, ‘$80 000 to $100 000’, ‘More than $100 000’ or ‘Unsure/prefer not to answer’ (values in Canadian dollars). Responses were collapsed and recoded into five categories: ‘≤20 000’, ‘20 001–40 000’, ‘40 001–60 000’, ≥60 000’ and ‘prefer not to answer/missing’. Possible responses for ‘What is the highest level of education you have received?’ were ‘No schooling’, ‘Elementary’, ‘Secondary’, ‘Community college/technical college’, ‘University’, ‘Graduate university’ and ‘Prefer not to answer’. Responses were collapsed and recoded into four categories: ‘secondary or less’, ‘college diploma’, ‘university or graduate degree’ and ‘prefer not to answer/missing’.

### Diet

Children’s diet was assessed using the YAQ. Data from the YAQ were used to derive a score from the Diet Quality Index-International (DQI), a score from 0 to 100 ranking children’s diet quality based on adequacy, balance, moderation and variety^(^ ^25^ ^)^. DQI was divided into tertiles and treated as a categorical variable to generate meaningful comparisons and regression coefficients that are easy to interpret. Data from the YAQ were also used to assess children’s adherence to the recommendation about added sugars outlined by the WHO in the free sugars guideline^(^ ^26^ ^)^ as well as the recommendation for saturated fats derived from the US Department of Agriculture Dietary Guidelines for Americans^(^ ^27^ ^)^. Both these recommendations suggest less than 10 % of total energy from each of added sugar and saturated fat consumption. Total energy contributed by added sugar from snacks and sugar-sweetened beverages and by saturated fat was calculated and divided by total energy intake to determine if children met or did not meet each guideline. Total energy intake was also derived from responses to the YAQ.

### Potential confounders

Potential confounders included child’s sex and region of residence as determined by the home survey. Heights and weights to determine body weight status were measured directly by research assistants during visits to schools to administer the child surveys. Body weight status was defined using International Obesity Task Force age- and sex-specific cut-offs^(^ ^28^ ^)^. Self-reported adherence to other lifestyle recommendations including sleep, screen time and physical activity were included as potential confounders. Sleep duration was assessed using usual bed and wake times of children reported by parents, screen time was self-reported by children on the student survey, and physical activity was assessed using the Physical Activity Questionnaire for Children and Youth (PAQ-C), which generates a score of 0–5 to describe children’s levels of moderate-to-vigorous physical activity^(^ ^29^ ^)^. Adherence to sleep recommendations was assessed using the National Sleep Foundation guideline for this age group, which is 9–11 h^(^ ^30^ ^)^. Adequate physical activity was assessed using developed PAQ-C cut-offs that align with optimum minimal cardiorespiratory health for children, found to be a score of 2·9 for girls and 2·7 for boys^(^ ^31^ ^)^. Adherence to screen time recommendations was assessed using the Canadian Sedentary Behaviour Guidelines^(^ ^32^ ^)^ which recommend less than 2 h of screen time daily for children of this age group.

### Academic achievement

Results from mandatory, standardized exams for all children were provided by the Nova Scotia Department of Early Education and Child Development. Test scores were provided as dichotomous and coded as either ‘meeting expectations’ and ‘not meeting expectations’ as per standardized provincial criteria in the subjects of Reading, Writing and Mathematics. The Nova Scotia Department of Early Education and Childhood Development, who administers these exams, provides rubrics for the exams to determine if children are meeting or not meeting provincial expectations. Further information on this process can be found elsewhere (http://plans.ednet.ns.ca/about-plans).

### Analytic approach

All analyses were weighted for non-response to represent provincial estimates of the grade 5 student population in Nova Scotia^(^ ^33^ ^)^. Parents provided the postal code of their household; using these data, the neighbourhood-level income of participants was compared with that of non-participants using Canadian census data. It was found that non-response was higher among lower-income neighbourhoods in Nova Scotia and weights were calculated to account for this disproportionate non-response^(^ ^33^ ^)^. We applied mixed-effects models due to the clustering of students within schools. Univariable logistic regression was first used to assess the associations between food security status and children’s academic achievement, dichotomized into meeting or not meeting expectations for mathematics, reading and writing. The full multivariable model included all three variables adjusted for all covariates of interest: socio-economic indicators, child’s diet, body weight status, meeting of physical activity, sleep and screen time recommendations, child’s sex and region of residency. All analyses were conducted using the statistical software package Stata IC version 14.1.

## Results

The prevalence of varying levels of household food insecurity (excluding missing responses) was 16·9 %, with 9·8 % of these households reporting low food security and the remaining 7·1 % reporting very low food security. Twenty per cent of households reported an income less than $CAN 20 000. Meanwhile, 87·4, 89·1 and 70·6 % of students met expectations for reading, writing and mathematics, respectively. Of children who were food insecure, children had a median DQI of 62·2 (where 60 points is a cut-off for good diet quality^(^ ^25^ ^)^); 54·3 and 62·6 % of students met the recommendation for saturated fats and free sugars, respectively (Table 1). Participants who consented to participate in the survey but not to the linkage to academic data differed significantly in likelihood of experiencing household food insecurity from those who consented to the linkage. Participants who did not consent to the linkage were more likely to report experiencing household food insecurity than those who did consent to the linkage.

**Table 1:** Characteristics of grade 5 (10–11-year-old) students participating in the Children’s Lifestyle and School performance Study (CLASS) in Nova Scotia, Canada, 2011

	Total	Girls (52·8 %)	Boys (47·2 %)
Met expectations in reading (%)	87·4	90·1	84·4
Met expectations in writing (%)	89·1	92·6	85·1
Met expectations in mathematics (%)	70·6	69·5	71·2
Food security status (%)			
High food security	78·2	77·1	79·5
Low food security	9·2	9·6	8·7
Very low food security	6·7	7·3	6·0
Missing	5·9	6·0	5·8
Household income ($CAN; %)			
≤20 000	20·3	21·8	18·6
20 001–40 000	13·3	13·3	13·3
40 001–60 000	24·1	22·9	25·4
≥60 001	19·8	19·2	20·4
Prefer not to answer/missing	22·5	22·8	22·2
Parental education (%)			
Secondary or less	17·3	19·1	15·4
College diploma	38·4	37·4	39·6
University or graduate degree	36·3	35·1	37·7
Prefer not to answer/missing	8·0	8·5	7·3
Diet Quality Index, mean	61·7	61·9	61·5
sd	9·9	9·8	10·1
Meeting dietary recommendations (%)			
Saturated fat intake (<10 % total energy)	54·3	55·8	47·3
Free sugars intake (<10 % total energy)	62·6	63·2	61·9
Weight status (%)			
Normal weight	65·3	66·4	64·1
Overweight or obese	34·7	33·6	35·9
Meeting physical activity cut-off (score >2·7 for girls, >2·9 for boys; %)	76·7	73·5	79·6
Meeting screen time recommendation (<2 h/d; %)	77·7	76·5	79·0
Meeting sleep recommendation (9–11 h/d; %)	91·1	90·7	91·5
Age (years), mean	11·0	11·0	11·0
sd	0·4	0·4	0·4
Rural residence (%)	30·3	30·3	30·4

Of students who met expectations in reading who had complete food insecurity information, 84·1 % were experiencing high food security, 9·2 % were experiencing low food security and 6·7 % were experiencing very low food security. The same percentages applied for children who met expectations in writing. Of students who met expectations in mathematics who had complete food insecurity information, 83·9 % were experiencing high food security, 9·3 % were experiencing low food security and 6·8 % were experiencing very low food security.

Conversely, of students who were experiencing high food security, 88·5, 90·2 and 73·5 % met expectations in reading, writing and mathematics, respectively. Of students who were experiencing low food security, 83·9, 85·7 and 61·8 % met expectations in reading, writing and mathematics, respectively. Of students who were experiencing very low food security, 77·7, 82·1 and 50·6 % met expectations in reading, writing and mathematics, respectively.

Unadjusted analyses between food insecurity and academic achievement are presented in Table 2. In unadjusted analyses, food insecurity had a strong, negative relationship with test scores in all three academic subjects (reading, writing and mathematics). Experiencing low food security was associated with 0·70, 0·65 and 0·59 times the odds of meeting expectations in reading, writing and mathematics, respectively. Very low food security was associated with 0·48, 0·52 and 0·38 times the odds of meeting expectations for reading, writing and mathematics, respectively.

**Table 2:** Relationship of food security status with the odds of meeting expectations on standardized exams among grade 5 (10–11-year-old) students participating in the Children’s Lifestyle and School performance Study (CLASS) in Nova Scotia, Canada, 2011

	Reading			Writing			Mathematics		
Characteristic	OR	95 % CI	*P* value	OR	95 % CI	*P* value	OR	95 % CI	*P* value
Food security status									
High food security (reference)	1·00	–	–	1·00	–	–	1·00	–	–
Low food security	**0**·**70**	**0**·**51, 0**·**96**	**0**·**03**	**0**·**65**	**0**·**44, 0**·**96**	**0**·**03**	**0**·**59**	**0**·**45, 0**·**78**	**<0**·**001**
Very low food security	**0**·**48**	**0**·**34, 0**·**68**	**<0**·**001**	**0**·**52**	**0**·**35, 0**·**76**	**0**·**001**	**0**·**38**	**0**·**29, 0**·**51**	**<0**·**001**

All presented OR are univariate; significant results are indicated in bold font.

Table 3 presents the fully adjusted models, where the relationship between food insecurity and academic achievement is adjusted for socio-economic status, diet, weight status, meeting recommendations for physical activity and screen time, child’s sex and region of residence. Considering these influences, children who resided in households reporting very low food security had 0·65 times the odds of meeting expectations in reading and 0·62 times the odds of meeting expectations in mathematics (OR=0·65; 95 % CI 0·44, 0·96 and OR=0·62; 95 % CI 0·45, 0·86, respectively; Table 3). Socio-economic status, including household income and parental level of education, and diet quality continued to have independent, significant associations with academic achievement while considering all relevant confounders and measures of food insecurity. In addition, meeting the recommendation for added sugars was strongly and positively associated with increased likelihood of meeting expectations in all three academic subjects considering all other covariates. Children who met the added sugars recommendation were 1·46, 1·41 and 1·25 times more likely to meet expectations in reading, writing and mathematics, respectively (Table 3). Male sex was strongly negatively associated with likelihood of meeting expectations in reading (OR=0·58; 95 % CI 0·47, 0·70) and writing (OR=0·45; 95 % CI 0·37, 0·56), but showed no association with likelihood of meeting expectations in mathematics (Table 3). Children living in urban residences had 0·70 times the odds of meeting expectations in mathematics compared with children in rural residences (OR=0·70; 95 % CI 0·56, 0·87; Table 3). None of body weight status, meeting recommendations for saturated fat, physical activity and sleep had any significant associations with meeting expectations in any academic subject.

**Table 3:** Relationship of food security status, diet, socio-economic indicators and relevant confounders with odds of meeting expectations on standardized exams among grade 5 (10–11-year-old) students participating in the Children’s Lifestyle and School performance Study (CLASS) in Nova Scotia, Canada, 2011

	Reading			Writing			Mathematics		
Characteristic	OR	95 % CI	*P* value	OR	95 % CI	*P* value	OR	95 % CI	*P* value
Food security status									
High food security (reference)	1·00	–	–	1·00	–	–	1·00	–	–
Low food security	0·99	0·69, 1·41	0·96	0·86	0·58, 1·29	0·47	0·90	0·66, 1·22	0·50
Very low food security	**0**·**65**	**0**·**44, 0**·**96**	**0**·**03**	0·73	0·47, 1·12	0·15	**0**·**62**	**0**·**45, 0**·**86**	**0**·**004**
Household income ($CAN)									
≤20 000 (reference)	1·00	–	–	1·00	–	–	1·00	–	–
20 001–40 000	1·17	0·84, 1·63	0·36	1·40	0·98, 2·01	0·07	**1**·**37**	**1**·**07, 1**·**73**	**0**·**01**
40 001–60 000	1·25	0·86, 1·81	0·25	1·43	0·96, 2·14	0·08	**1**·**33**	**1**·**04, 1**·**70**	**0**·**03**
≥60 001	**1**·**90**	**1**·**24, 2**·**90**	**0**·**003**	**1**·**95**	**1**·**21, 3**·**17**	**0**·**007**	**1**·**85**	**1**·**38, 2**·**50**	**<0**·**001**
Parental education									
Secondary or less (reference)	1·00	–	–	1·00	–	–	1·00	–	–
College diploma	1·25	0·94, 1·66	0·12	1·16	0·84, 1·60	0·37	**1**·**33**	**1**·**07, 1**·**65**	**0**·**01**
University or graduate degree	**1**·**84**	**1**·**34, 2**·**52**	**<0**·**001**	1·31	0·92, 1·86	0·14	**2**·**38**	**1**·**89, 2**·**98**	**<0**·**001**
Diet Quality Index, tertiles									
Low (reference)	1·00	–	–	1·00	–	–	1·00	–	–
Medium	**1**·**44**	**1**·**09, 1**·**91**	**0**·**01**	**1**·**59**	**1**·**19, 2**·**13**	**0**·**002**	**1**·**23**	**0**·**99, 1**·**54**	**0**·**06**
High	**1**·**76**	**1**·**28, 2**·**43**	**0**·**001**	**1**·**66**	**1**·**15, 2**·**41**	**0**·**007**	**1**·**53**	**1**·**16, 2**·**20**	**0**·**003**
Meets recommendation for saturated fat	1·05	0·82, 1·34	0·71	0·97	0·74, 1·27	0·81	0·80	0·64, 1·01	0·06
Meets recommendation for free sugars	**1**·**46**	**1**·**17, 1**·**82**	**<0**·**001**	**1**·**41**	**1**·**13, 1**·**65**	**0**·**002**	**1**·**25**	**1**·**04, 1**·**49**	**0**·**02**
Weight status									
Normal weight (reference)	1·00	–	–	1·00	–	–	1·00	–	–
Overweight or obese	1·05	0·82, 1·34	0·12	0·83	0·85, 1·38	0·17	0·98	0·82, 1·18	0·85
Meets physical activity cut-off	1·03	0·80, 1·34	0·80	1·08	0·85, 1·38	0·51	1·11	0·93, 1·33	0·23
Meets screen time recommendation	0·95	0·75, 1·21	0·69	1·30	1·00, 1·70	0·049	1·01	0·82, 1·24	0·94
Meets recommendation for sleep	1·41	0·89, 2·24	0·15	1·53	1·01, 2·32	0·046	1·46	0·99, 2·14	0·19
Sex									
Boy	**0**·**58**	**0**·**47, 0**·**70**	**<0**·**001**	**0**·**45**	**0**·**37, 0**·**56**	**<0**·**001**	1·12	0·95, 1·31	0·18
Girl (reference)	1·00	–	–	1·00	–	–	1·00	–	–
Urban residence	1·09	0·85, 1·38	0·50	0·84	0·64, 1·10	0·21	**0**·**70**	**0**·**56, 0**·**87**	**0**·**001**

All OR are adjusted for all covariates listed in this table as well as energy intake; significant results are indicated in bold font.

## Discussion

The present study revealed that very low food insecurity in grade 5 students is negatively associated with their achievement on standardized exams written in grade 6 in Nova Scotia, Canada. These findings are important given the persistently high prevalence of food insecurity in Nova Scotia^(^ ^4^ ^)^. In addition, there are few studies that have investigated the association between food insecurity and academic achievement in Canada, where household food insecurity is a prevalent and important concern. The findings from the present cross-sectional study prospectively linked to academic achievement results suggest that longitudinal, population-based study of the association between food insecurity and academic achievement in Canada is merited.

The present findings about the negative association between food insecurity and academic achievement for students in a Westernized context are consistent with existing literature^(^ ^11^ ^,^ ^12^ ^,^ ^15^ ^,^ ^34^ ^)^. Using cross-sectional data from the National Health and Nutrition Examination Survey, Alaimo *et al*. found that children aged 6–11 years in the USA experiencing food insufficiency in the household had decreased scores in both reading and arithmetic, and were also more likely to repeat a grade^(^ ^35^ ^)^. Jyoti *et al*., using data from a large, longitudinal study of American children, found that the presence of food insecurity resulted in impaired performance in reading and mathematics as well as consistent delays in reading ability throughout the schooling trajectory^(^ ^11^ ^)^. Studies investigating the association between food insecurity and academic achievement in Westernized contexts have occurred predominantly in the USA. The present study contributes findings from a Canadian context where studies are lacking. The negative association between food insecurity and schooling outcomes has also been observed in children in pre-school as well as in university students who are experiencing food insecurity, suggesting a consistent, negative association between food insecurity and academic achievement throughout the life trajectory^(^ ^36^ ^,^ ^37^ ^)^. This negative association is also consistently seen in non-Western contexts^(^ ^13^ ^,^ ^14^ ^,^ ^38^ ^)^.

Food insecurity has been found to be negatively associated with outcomes that may contribute to poor academic achievement in school-aged children, including poor psychosocial outcomes, mental health and cognitive development. Studies have shown that children experiencing household food insecurity are at risk of behavioural and emotional issues including affecting their ability to be engaged in school^(^ ^39^ ^)^. Children from food-insecure households are less likely to get along with peers, are at higher risk of hyperactivity and are more likely to see a psychologist during their formative years^(^ ^35^ ^,^ ^40^ ^)^. Children from food-insecure households are also more likely to have high rates of absenteeism and tardiness^(^ ^13^ ^,^ ^41^ ^)^. Parents in food-insecure households are also more likely to experience high levels of stress and adverse mental health, which may influence their ability to care for and support their children in academic pursuits^(^ ^42^ ^,^ ^43^ ^)^. Young children who are experiencing food insecurity may also experience negative cognitive skill development, laying the foundation for poor academic achievement when they enter formal schooling^(^ ^10^ ^,^ ^15^ ^)^.

Food insecurity has also been shown to compromise dietary intake potentially resulting in malnutrition and, subsequently, poor academic achievement^(^ ^44^ ^–^ ^46^ ^)^. The dearth of studies evaluating the mediating effect of diet on the relationship between food insecurity and academic achievement has been identified as a major gap in the literature^(^ ^12^ ^)^. As cross-sectional study designs, as used in the present study, are not considered to be appropriate for mediation analysis^(^ ^47^ ^)^, we attempted to evaluate the association between food insecurity and academic achievement outside its association via diet by adjusting for dietary indicators, at risk of overadjustment bias^(^ ^48^ ^)^. Despite a potential bias towards the null, very low food security had a strong, negative association with academic achievement despite a strong, independent association of higher diet quality with academic achievement. While this analysis cannot completely quantify the direct effects of food insecurity with academic achievement, it provides insight on the potential mediating effect of diet. Although the inclusion of diet in the full model attenuates the association between food insecurity and academic achievement, it does not completely attenuate the strong negative association between very low household food insecurity and academic achievement.

The current study has several strengths. First, it draws on a large, population-based sample of Canadian children with data linked to objective measures of academic achievement. The breadth of the data from this sample allowed us to consider multiple important factors in our analysis of food insecurity and academic achievement that have been identified as important considerations in analyses of this relationship. The study utilized a widely used and validated measure of household food insecurity. We have corrected for potential non-response bias through our analyses. The study also has several important limitations. The study design employed does not allow causal inference of the relationship between food insecurity and academic achievement. Adjustment for diet quality may potentially have biased estimates of the association between food insecurity and academic achievement. The six-item HFSSM does not measure child food insecurity, and as such we could not infer if the child who participated in the study was experiencing food insecurity to the same degree as other members of a food-insecure household. Although children live in a household where food insecurity is experienced, they may not be hungry or experience a compromised diet. Further study of this objective using the long-form HFSSM, or another tool that can identify if children are experiencing hunger through food insecurity, is necessary. In addition, we found that participants in the survey who did not consent to the academic linkage were more likely to be experiencing household food insecurity compared with those who did. Given this, Nova Scotian students experiencing household food insecurity are under-represented by our sample and our estimates may be underestimating the association between household food insecurity and academic achievement. Although the YAQ is a validated questionnaire, similarly to any subjective or invasive dietary measurement, it may still be prone to bias. Finally, it is possible that residual confounding may be affecting results.

Food security is an important issue that has shown no signs of decreasing in Nova Scotia and in Canada^(^ ^4^ ^)^. There are presently no provincial or federal initiatives in place that have the explicit goal of reduction of food insecurity among Canadians^(^ ^49^ ^)^. Findings from the current study suggest that the presence of food insecurity in a child’s household may compromise his/her academic achievement. Further research to elucidate a causal link between food insecurity and academic achievement in Canadian children and youth is merited.
